# Development and Preliminary Validation of the Lovebird Scale

**DOI:** 10.3390/bs14090747

**Published:** 2024-08-26

**Authors:** Sara Cloonan, Lara Ault, Karen L. Weihs, Richard D. Lane

**Affiliations:** 1 Department of Psychology, University of Georgia, Athens, GA 30605, USA; 2 Department of Social Sciences, Saint Leo University, Saint Leo, FL 33574, USA; lara.ault@saintleo.edu; 3 Department of Psychiatry, College of Medicine, University of Arizona, Tucson, AZ 85721, USA; weihs@psychiatry.arizona.edu (K.L.W.); lane@psychiatry.arizona.edu (R.D.L.)

**Keywords:** romantic relationships, relationship quality, romantic love, lovebirds, psychometrics

## Abstract

The term “lovebirds” is often used to describe the loving behaviors and interactions between two romantic partners, but what specific processes distinguish these flourishing lovebird relationships from other committed but “numbed” relationships? The present study aimed to address this knowledge gap through the development and preliminary validation of the Lovebird Scale. The Lovebird Scale describes the thoughts, feelings, behaviors, and habits that constitute and maintain relationship flourishing, which in turn could promote aspects of individual flourishing such as positive affect. We conducted three studies using data collected from 996 English-speaking U.S. adults (64.2% Female, *M* = 39.2 years old) who reported being in a romantic relationship for at least six months (*M* = 11.2 years). In Study 1, we conducted an exploratory factor analysis to determine the underlying factor structure. In Study 2, confirmatory factor analyses revealed a three-factor model nested within a higher-order factor representing lovebird relationships. In Study 3, we cross-validated the higher-order structure, examined the construct validity of the scale, and explored associations between the Lovebird Scale and affective state. Finally, we discuss how the Lovebird Scale contributes to the growing field of positive relationship science as well as conceptual and clinical implications of the scale.

## 1. Introduction

The importance of romantic relationships for health and well-being is well documented. Individuals in committed relationships tend to report better mental and physical health, engage in fewer risky behaviors, and have lower morbidity and mortality rates [1,2,3,4]. Merely being in a long-term committed relationship does not guarantee these benefits; rather, they are largely dependent on the quality of the relationship [3,5]. Thus, building and maintaining high relationship quality over time appear key to attaining the benefits of being in a romantic relationship. In order to do this, however, there is a need to (1) identify the properties that constitute and maintain such high-quality relationships and (2) determine how these properties contribute to aspects of individual health and well-being.

Previous research has primarily focused on the presence (or absence) of negative processes that diminish relationship quality largely because these negative processes tend to have a greater impact on relationship stability and health outcomes [6,7]. For example, Gottman [8] found it takes five positive behaviors to counterbalance the impact of one negative behavior on participants’ moods during relationship conflict. While understanding the consequences of negative relationship processes has helped illustrate the importance of romantic relationships for health and well-being, this focus paints an incomplete picture of relationship functioning. Instead, an absence of dysfunction may mean an absence of pain but not the presence of positive benefits [9]. Fincham and Beach (2010) [7] have referred to functioning relationships that lack such rewards as “numbed” relationships. While these numbed relationships may be long-lasting, partners’ commitment might be motivated by a desire to avoid relationship dissolution rather than a desire to approach relationship maintenance [9]. Beyond screening for low-satisfied couples, however, there is also a need to delineate between those who are moderately satisfied but numbed and those who are highly satisfied with their relationships. Differentiating between these types of relationships is key to understanding how to facilitate optimal relationship functioning rather than preventing relationship suffering.

To address this, recent work has emphasized the construct of *relationship flourishing*. At the individual level, flourishing is characterized by high levels of positive emotion and psychosocial functioning [10]; similarly, relationship flourishing aims to capture the positive relationship processes that contribute to “a sense that their life as a couple is a life well lived” [7]. These processes can include, but are not limited to, emotional connection, partner support, forgiveness, acceptance, trust, respect, positive affect, satisfaction, commitment, and love [7]. Over the past decade, scholars have developed several models to further our understanding of relationship flourishing. For example, the Strong Relationality Model of Relationship Flourishing (SRM) is centered on the idea that “Ethical Responsiveness” (i.e., viewing one’s partner as an “Other” versus an object) motivates partners to engage in pro-relationship behaviors called “Responsible Actions” (e.g., gratitude, support, affection), which then facilitates other forms of “Relational-Connectivity” (e.g., intimacy, belongingness, mutual friendship) [11]. Wood, et al. [12] provide empirical support for the SRM in their longitudinal study, finding that perceived partner support mediated the impact of individual stress on gratitude–recognition (i.e., “Responsible Actions”) 12 months later, which then resulted in greater intimacy (i.e., “Relational-Connectivity”) between partners at 24 months. In addition to relational-connectivity, Galovan, et al. [13] also highlight the important role of dispositional virtues (e.g., viewing one’s partner as being “other-centered” versus “self-centered”, humble, compassionate, and positive), ethical behaviors (e.g., making time for one another, performing random acts of kindness for each other), and a greater focus on “we” rather than “I” in creating and maintaining flourishing relationships. In sum, these findings show that there is more to relationship quality than just perceptions of satisfaction, particularly within flourishing relationships [13,14,15].

The emphasis on negative relationship processes that, until recently, has dominated the literature has largely influenced the way we assess relationship quality [7,15]. Specifically, existing measures tend to operationalize relationship quality as a unidimensional construct with a single bipolar dimension ranging from extremely satisfied to extremely dissatisfied [7,16]. While previous research has justified the “conceptual simplicity” of existing scales to prevent misinterpretation and ambiguity [16], it is also possible that the global, unidimensional approach to measuring relationship quality fails to capture the intricacies of relationships that contribute to relationship flourishing [15]. However, several measures targeting aspects of flourishing relationships have been published in recent years. The Relationship Flourishing Scale (RFS) takes a eudemonic rather than hedonic approach to measuring relationship quality by assessing the degree of meaning, personal growth, relational giving, and goal sharing individuals derive from their relationship [15]. The Relational-Connectivity Scale (RCS) is based on the Strong Relationality Model of Relationship Flourishing (SRM) and aims to evaluate couples’ sense of belonging, mutual friendship, and intimacy [13]. While both measures offer valuable insight into specific facets of relationship flourishing, they are missing domains needed to assess relationship flourishing holistically, such as relational savoring, physical intimacy, responsiveness, self-disclosure, and positive affectivity. Thus, describing the specific behaviors, interactions, and feelings that contribute to the development and maintenance of long-lasting, flourishing relationships is key to improving both relational and individual well-being.

The present research aims to add to the field of relationship science through the development and validation of a multidimensional, self-report measure of relationship flourishing called the Lovebird Scale. We use the term “lovebirds” as it is often used colloquially to describe extremely affectionate and long-lasting romantic couples who are “in love” with each other, as opposed to just having loving feelings towards one another. Further, the term lovebirds captures the feelings of closeness and warmth in everyday interactions between partners that reinforce and further deepen their love for each other. Thus, the Lovebird Scale aims to bridge the gap between lay and academic understandings of lovebirds and relationship flourishing by developing a multifaceted scale that captures the specific behaviors, cognitions, and feelings that contribute to flourishing relationships, which differ from other long-term committed but numbed relationships. Further, the Lovebird Scale addresses key domains identified by previous scales (e.g., RFS, RCS) in addition to other constructs, such as savoring and responsiveness, to provide a broader understanding of relationship flourishing.

A secondary goal of this study is to examine positive and negative affectivity of individuals in flourishing relationships using the newly developed Lovebird Scale. Previous research has shown that flourishing individuals experience more positive affect than negative affect at a ratio of approximately 3:1 [17,18]; however, few studies have examined positive and negative affectivity within the context of flourishing relationships [7]. To address this, we hypothesized that the Lovebird Scale would be associated with more positive affect (PA) and less negative affect (NA). Further, we expected the Lovebird Scale to predict affective state above and beyond existing measures of relationship quality.

Through this research, we hope to further our understanding of what constitutes long-lasting, flourishing lovebird relationships as well as what makes them distinct from other committed but numbed relationships. Moreover, with a distinct measure of “above average” relationship quality, researchers could examine how lovebirds’ conflict resolution strategies, communication styles, and physical and emotional health may differ from those who are merely satisfied with their relationship.

## 2. Study 1

### 2.1. Materials and Methods

#### 2.1.1. Item Generation

Items on the initial Lovebird Scale were developed based on unstructured qualitative interviews with long-term romantic couples who described themselves as being lovebirds. Couples were recruited using convenience sampling from the community surrounding a public university in the Southwest United States. While unstructured interviews are more time-consuming and, thus, limit the number of interviews that can be conducted, an important strength of them is that participants are allowed to use their own words to describe their relationship and why they consider themselves to be lovebirds. The initial set of lovebird items were generated based on constructs identified in previous studies (e.g., goal sharing, mutual friendship, responsible actions, and intimacy) and on the themes and quotes taken from the interviews (e.g., prioritizing each other’s happiness, the “little things” their partner does for them, physical intimacy, and respect). To help distinguish lovebird relationships from other types of relationships, we developed another set of items aiming to capture committed but numbed relationships. In the present research, numbed relationships were characterized as those in which partners may be satisfied with their relationship but are not positively engaged as robustly as those in lovebird relationships (See Galovan, Carroll, Schramm, Leonhardt, Zuluaga, McKenadel and Oleksuik [13] description of “satisfied but less connected” relationships or Fincham and Beach’s (2010) [7] description of numbed relationships). Individuals in numbed relationships may not consider themselves to be deeply “in love” with each other and may be motivated to stay together for reasons other than their love for each other (e.g., financial reasons, children/dependents).

This first iteration of the Lovebird Scale contained 74 items, which were then reviewed by a panel of four relationship experts that included faculty members and clinicians from the psychology and psychiatry departments at a public university in the Southwest United States, who then excluded items that were redundant or were ambiguously written. The remaining 49 items formed the initial Lovebird Scale that was tested in Study 1. The participants were instructed to read each statement and choose the most appropriate response using a 7-point Likert scale ranging from 1 (*Strongly disagree*) to 7 (*Strongly agree*)*,* keeping their current partner and relationship in mind.

#### 2.1.2. Participants and Procedure

Recruitment and data collection for Study 1 occurred in June 2021. Participants were recruited via CloudResearch [19]. Participants needed to be at least 18 years old and currently involved in a romantic relationship for at least six months. Individuals who met the eligibility criteria and passed CloudResearch data security measures (i.e., ReCAPTCHA) were invited to complete an online Qualtrics survey (approximately 10 min) about romantic relationships. All participants provided electronic consent and were offered a monetary incentive for their participation. An institutional review board at a small, private Southeastern university reviewed and approved all aspects of this study.

A total of 552 participants completed Study 1. Twelve participants were excluded prior to analyses because they had missing data, reported a relationship length less than six months, or reported low relationship satisfaction. [20,21]. We chose to exclude participants with low relationship satisfaction because the goal of the scale is to differentiate between individuals in two types of satisfied relationships: those who may be in moderately satisfied but numbed relationships versus those in highly satisfied lovebird relationships. Low relationship satisfaction was defined as Relationship Assessment Scale (RAS) scores more than three standard deviations below the sample mean (i.e., total RAS scores < 11.08). The final analytic sample used for the EFA included *N* = 540. This resulted in an observation-to-item ratio of approximately 11, meeting previous recommendations for EFAs (between 5 and 10 observations per item) [22]. The participants were 39.9 years old (*SD* = 13.2), 60.6% female, 80.0% white, and 85.0% heterosexual. A total of 67.6% of the participants were married, and 91.3% currently lived with their partner. Their current relationship duration was 11.4 years (*SD* = 10.8 years) (see Table S1).

#### 2.1.3. Measures

In addition to the Lovebird Scale, the participants completed several measures of relationship quality as well as a demographic questionnaire that assessed age, biological sex, gender identity, racial/ethnic background, sexual orientation, and relationship length. Descriptive statistics and reliabilities for all scales used in Study 1 are in Table 1.

##### Relationship Assessment Scale

Relationship satisfaction was measured using the Relationship Assessment Scale (RAS), a 7-item scale designed to assess global relationship satisfaction [20]. Example items include “How well does your partner meet your needs?” and “How much do you love your partner?”. Cronbach’s alpha was reported to be α = 0.86 in the original sample [20].

##### Mutual Psychological Development Questionnaire

Perceived mutuality between partners was measured using the Mutual Psychological Development Questionnaire (MPDQ) [23]. The MPDQ is a 22-item self-report measure in which participants rate their own experience as well as perceptions of their partner’s experience when discussing something of importance to themselves or to their partner using a 6-point Likert scale. Example items include “be receptive” and “try to understand” for the self-subscale, and “respect point of view” and “see the humor in things” for the partner subscale. The MPDQ has excellent internal consistency (α = 0.92 for spouse/partner), test–retest reliability (α = 0.90), and construct validity [23].

##### Relationship Prototypes

We also included two investigator-developed relationship prototypes that described typical lovebird and numbed relationships to ensure that our conceptualization of lovebird and numbed relationships were being accurately captured by the Lovebird Scale. Each prototype consisted of approximately the same word count and semantic structure (*M* = 165.5 words). A sliding scale from 1 to 100 indicated how accurately each prototype described their current relationship. Both relationship prototypes can be found in Appendix B.

**Table 1:** Descriptive statistics and collections for Study 1 variables ^1^.

Variable	1	2	3	4	5	6	7	8
1. Mutuality	--							
2. Romance	0.72 ***	--						
3. Disconnect	−0.59 ***	−0.56 ***	--					
4. RAS	0.76 ***	0.69 ***	−0.71 ***	--				
5. MPDQ Self	0.57 ***	0.57 ***	−0.69 ***	0.64 ***	--			
6. MPDQ Partner	0.74 ***	0.60 ***	−0.65 ***	0.72 ***	0.69 ***	--		
7. Lovebird Prototype	0.72 ***	0.68 ***	−0.57 ***	0.81 ***	0.52 ***	0.65 ***	--	
8. Numbed Prototype	−0.28 ***	−0.35 ***	0.48 ***	−0.30 ***	−0.31 ***	−0.32 ***	−0.26 ***	--
*M*	5.50	5.48	2.93	28.9	4.77	4.51	75.8	46.3
*SD*	0.9	1.0	1.3	5.5	0.7	0.9	24.2	33.2
Range	5.15	4.80	6.00	23.0	3.60	4.44	100.0	100.0
Cronbach’s *a*	0.93	0.89	0.86	0.90	0.87	0.88	--	--

^1^ *N* = 540. RAS = Relationship Assessment Scale; MPDQ = Mutual Psychological Development Questionnaire. *** *p* < 0.001.

#### 2.1.4. Statistical Analyses

Data were analyzed using R Studio (version 2022.12.0+353) [Posit 24,R Core 25]). We conducted an exploratory factor analysis (EFA) using principal axis factoring (PAF) estimation with promax rotation to determine the underlying factor structure of the initial 49-item Lovebird Scale. Factorability of the data was determined using inter-item correlations (<0.80), the Kaiser–Meyer–Olkin (KMO) measure of sampling adequacy (>0.50), and the Bartlett’s test of sphericity (*p* < 0.05) [24]. We conducted a parallel analysis using the EFAtools R package to determine the appropriate number of factors to be extracted from the data [25]. The criteria to determine which items should be eliminated were (a) items with factor loadings less than 0.40; (b) items with cross-loadings greater than 0.20; (c) items with communalities less than 0.20; and (d) conceptual fit [24]. Cronbach’s alphas were also calculated to assess internal consistency, with values ≥ 0.70 considered acceptable [26]. Bivariate Pearson’s correlations were used to assess the preliminary convergent validity of the Lovebird Scale. Statistical significance for all analyses was set at the α = 0.05 level.

### 2.2. Results

The results from the KMO measure of sampling adequacy (0.97) and the Bartlett’s test of sphericity (*χ*^2^(1128) = 16,423.08, *p* < 0.001) suggested that an EFA was appropriate for the data. All 49 items had at least one inter-item correlation greater than 0.30, and no inter-item correlation pairs were greater than 0.80, further supporting the factorability of the data. Based on the results from the parallel analysis and Scree plot, we first tested a five-factor EFA solution, which explained 43% of the variance in the data. A three-factor solution yielded three distinct factors that had at least three items with factor loadings greater than 0.40 that also did not highly load onto the other factors. Thus, we decided to proceed with a three-factor solution. We removed items with low factor loadings or multiple high cross-loadings in a stepwise fashion, reevaluating the three-factor structure after each step, leaving 31 items. The final three-factor solution explained 46% of the variance in the items (Table 2).

Factor 1 was labeled “Mutuality”, Factor 2 was labeled “Romance”, and Factor 3 was labeled “Disconnect”. The Mutuality subscale captured the pro-relationship behaviors and social interactions that signal trust, acceptance, respect, and support. The Romance subscale reflected the behavioral and cognitive processes that facilitate feelings of love and passion between partners, such as physical intimacy and relational savoring. The Disconnect subscale included items that represented indifference and/or ambivalence towards the relationship. All three factors demonstrated high internal consistency (α = 0.86 to 0.93) and were significantly correlated with each other (*p* < 0.001). Bivariate correlations between the Lovebird Scale, relationship prototypes, and existing measures of relationship quality (RAS and MPDQ) provided preliminary evidence for the convergent validity of the scale. A summary of these correlations can be found in Table 1. We further examined the three-factor structure using confirmatory factor analyses in Study 2.

## 3. Study 2

### 3.1. Materials and Methods

#### 3.1.1. Participants and Procedure

Recruitment and data collection for Study 2 occurred in October 2021 using the same method and eligibility criteria as Study 1. Those who had not participated in Study 1 were invited to complete an online Qualtrics survey (approximately 15 min) about romantic relationships. All aspects of this study were IRB approved. All participants provided electronic consent prior to participation and were offered a monetary incentive for their participation.

A total of 223 participants completed Study 2. Removal of participants due to the exclusion criteria described in Study 1 resulted in a final analyzed sample of *N* = 215. The participants were 38.7 years old (*SD* = 11.6), 67% female, 84.7% white, and 86% heterosexual. A total of 61.4% of the participants were married, and 91.6% currently lived with their partner. The participants reported being in their current relationship for an average of 10.5 years (*SD* = 12.7 years). A summary of sample characteristics for Study 2 can be found in Table S1.

#### 3.1.2. Measures

In addition to the Lovebird Scale, RAS, MPDQ, relationship prototypes, and demographic questionnaire, the participants completed several other measures of relationship quality. Descriptive statistics and reliabilities for all scales used in Study 2 can be found in Table 3.

##### Perceived Relationship Quality Components Inventory

The Perceived Relationship Quality Components Inventory (PRQC) is an 18-item measure of relationship quality containing six subscales: Satisfaction (“How satisfied are you with your relationship?”), Commitment (“How committed are you to your relationship?”), Intimacy (“How connected are you to your partner?”), Trust (“How much can you count on your partner?”), Passion (“How passionate is your relationship?”), and Love (“How much do you love your partner?”). The PRQC has demonstrated high internal consistency (α = 0.78 to 0.96) in previous research [14].

##### Relationship Quality Scale

The Relationship Quality Scale (RQS) is a brief, 9-item measure of relationship quality that was developed using a diverse sample of individuals representing over 60 countries. Participants are asked to rate their degree of agreement with various statements regarding their current relationship. Example items include “This is the relationship I have always dreamed of” and “I think of my partner as my soulmate”. The RQS has demonstrated high internal consistency (α = 0.89) in previous research [27].

**Table 3:** Descriptive statistics and correlations for Study 2 variables ^1^.

Variable	1	2	3	4	5	6	7	8	9	10	11	12	13	14	15	16
1.Mutuality	--															
2.Romance	0.77 ***	--														
3.Disconnect	−0.71 ***	−0.69 ***	--													
4.Lovebird Composite	0.93 ***	0.88 ***	−0.89 ***	--												
5.RAS	0.83 ***	0.67 ***	−0.75 ***	0.84 ***	--											
6.MPDQ Self	0.69 ***	0.63 ***	−0.68 ***	0.75 ***	0.63 ***	--										
7.MPDQ Partner	0.79 ***	0.64 ***	−0.69 ***	0.79 ***	0.73 ***	0.76 ***	--									
8.PRQC Satisfaction	0.84 ***	0.72 ***	−0.71 ***	0.84 ***	0.89 ***	0.59 ***	0.72 ***	--								
9.PRQC Commitment	0.60 ***	0.62 ***	−0.58 ***	0.66 ***	0.62 ***	0.46 ***	0.45 ***	0.64 ***	--							
10.PRQC Intimacy	0.74 ***	0.79 ***	−0.67 ***	0.80 ***	0.79 ***	0.60 ***	0.65 ***	0.82 ***	0.55 ***	--						
11.PRQC Trust	0.74 ***	0.55 ***	−0.56 ***	0.69 ***	0.73 ***	0.49 ***	0.59 ***	0.74 ***	0.49 ***	0.59 ***	--					
12.PRQC Passion	0.46 ***	0.67 ***	−0.45 ***	0.56 ***	0.51 ***	0.44 ***	0.47 ***	0.58 ***	0.35 ***	0.72 ***	0.36 ***	--				
13.PRQC Love	0.73 ***	0.81 ***	−0.66 ***	0.80 ***	0.69 ***	0.59 ***	0.59 ***	0.72 ***	0.75 ***	0.74 ***	0.57 ***	0.52 ***	--			
14.RQS	0.86 ***	0.79 ***	−0.78 ***	0.90 ***	0.87 ***	0.67 ***	0.74 ***	0.87 ***	0.69 ***	0.79 ***	0.72 ***	0.54 ***	0.80 ***	--		
15.Lovebird Prototype	0.75 ***	0.73 ***	−0.68 ***	0.79 ***	0.77 ***	0.60 ***	0.62 ***	0.77 ***	0.56 ***	0.74 ***	0.54 ***	0.54 ***	0.75 ***	0.79 ***	--	
16.Numbed Prototype	−0.41 ***	−0.44 ***	0.60 ***	−0.54 ***	−0.46 ***	−0.44 ***	−0.45 ***	−0.45 ***	−0.27 ***	−0.45 ***	−0.32 ***	−0.42 ***	−0.40 ***	−0.49 ***	−0.40 ***	--
*M*	5.68	5.71	2.85	5.74	29.3	4.73	4.53	5.91	6.54	5.80	6.20	4.74	6.32	37.0	77.3	43.6
*SD*	1.1	0.9	1.3	1.0	5.2	0.7	0.9	1.2	0.9	1.3	1.1	1.7	1.1	6.6	26.8	37.9
Range	4.80	4.88	6.00	4.96	22.0	3.00	4.00	5.00	5.00	6.00	5.00	6.00	5.00	29.0	100	100
Cronbach’s *a*	0.92	0.87	0.87	0.95	0.90	0.86	0.89	0.96	0.95	0.89	0.89	0.93	0.92	0.92	--	--

^1^ *N* = 215. RAS = Relationship Assessment Scale; MPDQ = Mutual Psychological Development Questionnaire; PRQC = Perceived Relationship Quality Components Inventory; RQS = Relationship Quality Scale. *** *p* < 0.001.

#### 3.1.3. Statistical Analyses

Data were analyzed using R Studio (version 2022.12.0+353) [Posit 24,R Core 25]. We conducted a series of confirmatory factor analyses (CFAs) with robust maximum likelihood estimation (MLR) using the R package *lavaan*, version 0.6-18 [28]. We first tested the model proposed by the EFA in Study 1, followed by a one-factor model and an orthogonal model to examine any improvements in model fit. Factor variances were fixed to 1, and factor loadings were allowed to be freely estimated. Consistent with previous recommendations, overall model fit was evaluated using the following model fit indices: root-mean-square error of approximation (RMSEA) ≤ 0.06, comparative fit index (CFI) ≥ 0.90, standardized root-mean-square residual (SRMR) ≤ 0.08, and chi-square/degrees of freedom ratio (*χ^2^*/*df*) ≤ 3 [24,29,30]. We consulted modification indices to determine if there were any empirically and/or theoretically reasonable modifications that could be made to improve overall model fit. Once the best fitting model was identified, we examined a higher-order model in which the subscales of the Lovebird Scale were nested within a second-order factor representing lovebird relationships globally. Cronbach’s alphas were also calculated to assess internal consistency, with values ≥ 0.70 considered acceptable [26]. We tested the convergent validity of the Lovebird Scale by examining bivariate Pearson’s correlations. Statistical significance for all analyses was set at the α = 0.05 level.

### 3.2. Results

The results from the CFA on the EFA model were modest (*χ*^2^(431) = 875.9, *p* < 0.001; *χ*^2^/*df* = 2.03; CFI = 0.857; SRMR = 0.062; RMSEA = 0.069, 95% CI [0.063, 0.075], *p* < 0.001) but significantly better than the orthogonal (*χ*^2^(434) = 1223.4, *p* < 0.001; *χ*^2^/*df* = 2.82; CFI = 0.737; SRMR = 0.316 RMSEA = 0.092, 95% CI [0.087, 0.097], *p* < 0.001) and single-factor models (*χ*^2^(434) = 1002.3, *p* < 0.001; *χ*^2^/*df* = 2.82; CFI = 0.811; SRMR = 0.078; RMSEA = 0.078, 95% CI [0.072, 0.084], *p* < 0.001), supporting a multidimensional model with correlated factors. Based on modification indices, we made several revisions to the original EFA model in an incremental fashion: item 12 (“My partner and I have recurring problems that we can’t get past”) was moved from Mutuality to Disconnect, item 37 (“My partner and I go through life savoring moments together”) was moved from Romance to Mutuality, and item 13 (“We are each other’s best friend”) was moved from Mutuality to Romance; item 14 (“My partner and I know how to make each other laugh, even on our bad days”) was removed from the model due to high cross-loadings on Mutuality and Romance; and items 4, 19, 26, and 27 were removed from the model due to low loadings on their assigned factors. The revised 26-item model produced acceptable model fit statistics (*χ*^2^(296) = 485.6, *p* < 0.001; *χ*^2^/*df* = 1.64; CFI = 0.925; SRMR = 0.055; RMSEA = 0.055, 95% CI [0.047, 0.062], *p* = 0.162). Standardized regression weights ranged from 0.556 to 0.882 (*p* <.001), and all item variances were positive (i.e., no Heywood cases). Based on these results, we retained 26 items for the final Lovebird Scale (Appendix A). The three subscales exhibited high internal consistency, with Cronbach alphas ranging from 0.87 to 0.92.

**Figure 1:**
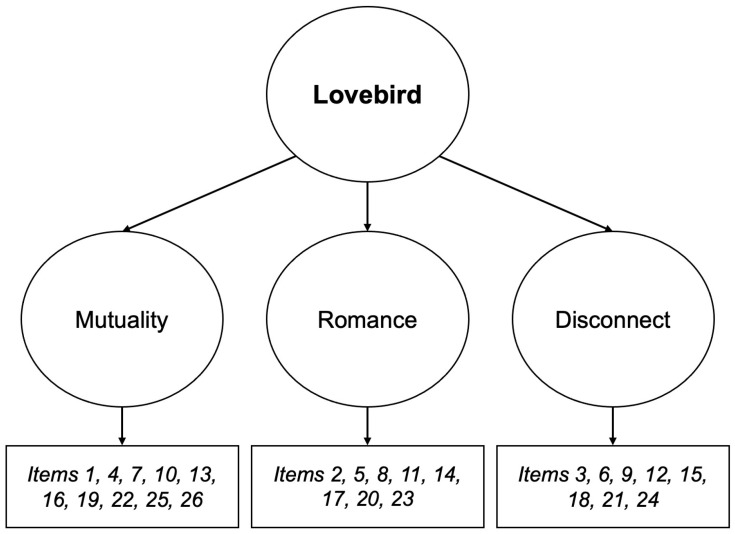
Higher-order factor structure of the Lovebird Scale. Item numbers are based on those listed in the final version of the Lovebird Scale found in Appendix A.

To account for high inter-factor correlations, we tested a higher-order model in which the three factors were nested within one higher order factor representing lovebird relationships (Figure 1). Although model fit indices for the higher-order model in this sample were slightly less than those of the original three-factor model and the recommended cut-offs, they were still in an acceptable range [30]. Moreover, the higher-order Lovebird factor accounted for a large portion of the variance among the first-order factors, with *R*^2^ values ranging from 0.746 to 0.871. The higher-order Lovebird factor also had high internal consistency, with a Cronbach’s alpha of 0.95.

Convergent validity for the revised Lovebird Scale was tested using correlations with the relationship prototypes and existing measures of relationship quality (RAS, MPDQ, PRQC, and RQS). All correlations for the three subscales were statistically significant (*p* < 0.001) and in the expected direction. Composite lovebird scores were created by reverse scoring items on the Disconnect subscale and then calculating the average of all three subscales. As expected, the composite lovebird scores were significantly and positively correlated with the RAS, MPDQ, PRQC, and RQS (*p* < 0.001). A summary of correlations between all Study 2 variables can be found in Table 3. We cross-validated the higher-order model and investigated the construct validity in Study 3.

## 4. Study 3

### 4.1. Materials and Methods

#### 4.1.1. Participants and Procedure

Recruitment and data collection for Study 3 occurred in December 2021 using the same method and eligibility criteria as Study 1. Those who had not participated in Study 1 or 2 were invited to complete an online Qualtrics survey (approximately 30 min) about romantic relationships. Institutional review board approval, collection of electronic consent, and a monetary incentive were identical to Studies 1 and 2. A total of 252 participants completed Study 3. Eleven participants were removed based on the exclusion criteria described in Study 1 and/or scored over the sample mean Infrequency score (*M* = 1.1), resulting in a final analytic sample of *N* = 241. On average, the participants were 39.1 years old (*SD* = 10.2), 69.7% female, 83% white, and 86.7% heterosexual. Seventy percent of the participants were married, and 92.9% currently lived with their partner. The participants reported being in their current relationship for an average of 11.7 years (*SD* = 11.2 years). A summary of sample characteristics for Study 3 can be found in Table S1.

#### 4.1.2. Measures

In addition to the Lovebird Scale, RAS, MPDQ, relationship prototypes, and demographics questionnaire, the participants completed several other measures of relationship quality, attachment style, and affective state. Since the survey for Study 3 was longer than the first two surveys, we also included a six-item attention check measure (Infrequency Scale). Descriptive statistics and reliabilities for all scales used in Study 3 are in Table 4.

##### Dyadic Adjustment Scale

The Dyadic Adjustment Scale-32 (DAS-32) is a 32-item scale designed to assess relationship quality in cohabiting or married couples [31]. The DAS-32 consists of ordinal, Likert, and dichotomous scales, with total scores ranging from 0 to 151. The DAS-32 has also demonstrated acceptable internal consistency and construct validity in previous research (α = 0.58 to 0.96; *M* = 0.915; 95% CI [0.906, 0.922]) [31,32].

##### Adult Attachment Scale

Anxious and avoidant attachment were measured using the revised 18-item Adult Attachment Scale (AAS). Statements describe varying degrees of comfort with closeness and intimacy as well as fear of rejection within close relationships [33]. The revised AAS has demonstrated acceptable reliability in previous research (α = 0.80 to 0.83) [33].

##### Positive and Negative Affect Schedule

Affective state was measured using the Positive and Negative Affect Schedule (PANAS), a 20-item self-report measure containing 10 items representing PA (e.g., “Enthusiastic”, “Attentive”) and 10 items representing NA (e.g., “Irritable”, “Upset”) [34]. Participants are asked to indicate the extent to which they have felt each affective state over the past week. The PANAS has also demonstrated high internal consistency in previous research (PA α = 0.89, 95% CI [0.88–0.90]; NA α = 0.85, 95% CI [0.84–0.87]) [35].

##### Infrequency Scale (Attention Check)

Previous research suggests including attention check measures in online surveys to ensure participants are paying attention to the questions and are answering honestly [36]. Thus, a brief, 6-item Infrequency Scale was also included in the assessment battery as an attention check measure. Example items include “I enjoy visiting London, Wisconsin” and “I once rode my bicycle from New York City to San Diego”. Participants who scored over the sample mean Infrequency score (*M* = 1.1) were removed prior to analysis.

**Table 4:** Descriptive statistics and correlations for Study 3 variables ^1^.

Variable	1	2	3	4	5	6	7	8	9	10	11	12	13	14
1.Mutuality	--													
2.Romance	0.72 ***	--												
3.Disconnect	−0.70 ***	−0.66 ***	--											
4.Lovebird Composite	0.92 ***	0.86 ***	−0.89 ***	--										
5.RAS	0.82 ***	0.70 ***	−0.78 ***	0.86 ***	--									
6.MPDQ Self	0.60 ***	0.65 ***	−0.61 ***	0.69 ***	0.58 ***	--								
7.MPDQ Partner	0.73 ***	0.64 ***	−0.70 ***	0.78 ***	0.67 ***	0.74 ***	--							
8.DAS-32	0.70 ***	0.63 ***	−0.67 ***	0.75 ***	0.69 ***	0.59 ***	0.67 ***	--						
9.AAS Anxiety	−0.23 **	−0.13 *	0.34 ***	−0.28 ***	−0.28 ***	−0.35 ***	−0.35 ***	−0.29 ***	--					
10.AAS Avoidant	−0.18 **	−0.17 **	0.17 **	−0.20 **	−0.18 **	−0.34 ***	−0.27 ***	−0.24 ***	0.46 ***	--				
11.PANAS Positive	0.20 **	0.34 ***	−0.18 **	0.25 ***	0.21 ***	0.38 ***	0.26 ***	0.20 **	−0.36 ***	−0.44 ***	--			
12.PANAS Negative	−0.08	−0.05	0.21 **	−0.14 *	−0.13	−0.25 ***	−0.20 **	−0.23 ***	0.45 ***	0.46 ***	−0.31 ***	--		
13.Lovebird Prototype	0.76 ***	0.74 ***	−0.71 ***	0.79 ***	0.84 ***	0.48 ***	0.61 ***	0.64 ***	−0.32 ***	−0.21 ***	0.23 ***	−0.11	--	
14.Numbed Prototype	−0.24 ***	−0.35 ***	0.42 ***	−0.37 ***	−0.32 ***	−0.30 ***	−0.28 ***	−0.32 ***	0.18 **	−0.06	−0.14 *	0.05	−0.32 ***	--
*M*	5.75	5.73	2.83	5.78	29.6	4.72	4.55	111.3	2.76	2.72	33.1	17.9	77.9	47.9
*SD*	1.0	0.9	1.2	1.0	5.2	0.7	0.8	22.0	1.0	0.8	7.9	7.6	24.0	39.4
Range	4.00	3.62	5.12	4.00	23.0	3.45	3.78	100.0	4.00	3.92	38.0	35.0	100.0	100.0
Cronbach’s *a*	0.92	0.85	0.86	0.94	0.91	0.86	0.88	0.95	0.88	0.90	0.92	0.93	--	--

^1^ *N* = 241. RAS = Relationship Assessment Scale; MPDQ = Mutual Psychological Development Questionnaire; DAS = Dyadic Adjustment Scale—Total; AAS = Adult Attachment Scale; PANAS = Positive and Negative Affect Schedule. * *p* < 0.05; ** *p* < 0.01; *** *p* < 0.001.

#### 4.1.3. Statistical Analyses

Data were analyzed with the statistical package and methods used in Study 2. We conducted confirmatory factor analyses (CFA) with robust maximum likelihood estimation (MLR) using the R package *lavaan* [28]. Model fit and internal consistency were evaluated using the same criteria used in Study 2. We tested the convergent validity of the Lovebird Scale by examining bivariate Pearson correlations with existing measures of relationship quality (RAS, MPDQ, DAS-32). We used the Fronell–Larcker criterion to assess discriminant validity, which states that the square root of the average variance extracted (AVE) of each latent variable must be greater than the correlation between the latent variable and other constructs, in this case, attachment style. Finally, we tested the incremental validity of the Lovebird Scale by estimating a series of regression models where both the Lovebird Scale, RAS, and DAS-32 were entered as simultaneous predictors of PA and NA. Statistical significance for all analyses was set at the α = 0.05 level.

### 4.2. Results

The higher-order model demonstrated acceptable model fit in our cross-validation analyses, although slightly lower than those observed in Study 2 (*χ*^2^(296) = 616.64, *p* < 0.001; *χ*^2^*/df* = 2.08; CFI = 0.885; SRMR = 0.061; RMSEA = 0.067, 95% CI [0.060, 0.074], *p* < 0.001). Standardized regression weights ranged from 0.469 to 0.833 (*p* < 0.001). The higher-order Lovebird factor accounted for a large portion of the variance among the first-order factors, with *R*^2^ values ranging from 0.818 to 0.845. The three subscales exhibited high internal consistency, with Cronbach alphas ranging from 0.85 to 0.92. The higher-order Lovebird factor also had high internal consistency, with a Cronbach’s alpha of 0.94.

We tested the convergent validity of the Lovebird Scale by examining correlations with the relationship prototypes and existing measures of relationship quality (RAS, MPDQ, and DAS-32). All correlations were statistically significant (*p* < 0.05) and in the expected direction. Correlations between composite lovebird scores and existing relationship quality measures ranged from 0.50 to 0.86, with the smallest correlation being with the DAS Consensus subscale and largest correlation being with the RAS and DAS Satisfaction subscale. A summary of correlations between all Study 3 variables can be found in Table 4.

We assessed discriminant validity using the Fronell–Larcker criterion. We first computed the AVE for the three subscales and the higher-order Lovebird factor, which ranged from 0.43 to 0.55. We then calculated the square root of each AVE and compared them with correlations with the AAS Anxiety and Avoidant subscales. We observed small but statistically significant correlations (*p* < 0.05) between the three Lovebird subscales, composite lovebird scores, and the AAS subscales, all in the expected directions. Importantly, these correlations were smaller than the square root of the AVE for each latent variable, providing initial evidence for the discriminant validity of the Lovebird Scale.

Prior to our tests of incremental validity, we examined associations between the Lovebird Scale and both PANAS subscales. As expected, composite lovebird scores, Mutuality, and Romance were significantly associated with greater PA (*p* < 0.01). Disconnect was significantly correlated with less PA and more NA (*p* < 0.05). Surprisingly, neither Mutuality nor Romance were significantly correlated with NA, but there was a small negative correlation between composite lovebird and NA (*r* = −0.14, *p* < 0.05). We then tested the incremental validity of the Lovebird Scale by regressing both PANAS subscales on composite lovebird scores while controlling for the RAS and DAS-32. The Lovebird Scale emerged as a significant and unique predictor of PA above and beyond the RAS and DAS-32 but not NA. In both sets of models, composite lovebird scores were positively associated to PA (RAS model: *β* = 2.86, *p* = 0.007; DAS-32 model: *β* = 2.28, *p* = 0.005). Composite lovebird scores were not significantly associated to NA when controlling for the RAS and DAS-32 (RAS model: *β* = −0.91, *p* = 0.392; DAS-32 model: *β* = 0.19, *p* = 0.809).

## 5. Discussion

The field of relationship science is increasingly striving to understand what contributes to optimal relationship functioning as opposed to dysfunction [7,15]. The results of the three studies reported here provide preliminary evidence for the construct validity of the Lovebird Scale, a new domain-specific measure of optimal relationship function. Its development is an important step for understanding the key factors that contribute to long-lasting, flourishing relationships, including the various behavioral, cognitive, and affective processes that occur within such relationships.

### 5.1. Psychometric Properties of the Lovebird Scale

We conducted three studies to develop and validate the Lovebird Scale using data collected from three independent samples comprised of approximately one-thousand individuals in long-term (i.e., ≥6 months) romantic relationships. Mutuality, Romance, and Disconnect were the factors that resulted from the exploratory and confirmatory factor analyses conducted in Studies 1 and 2. We also tested a higher-order factor structure with the three subscales nested within one overarching lovebird factor, consistent with the idea that relationship quality is a multidimensional construct with several semi-independent domains that contribute to overall relationship quality [14]. The overarching lovebird factor and the three moderately correlated subscales nested within it surpassed standard Cronbach’s alpha criteria (≥0.70) across all three studies, supporting the internal consistency of the scale. The Lovebird Scale also exhibited good convergent validity in all three studies, as observed through correlations with existing measures of relationship quality.

Mutuality within intimate relationships has previously been defined as the “modes of social interaction that facilitate participation in and growth through the relationship” [23]. In line with this definition, the Mutuality subscale within the Lovebird Scale captures the behaviors and interactions that reflect trust, respect, and responsiveness between partners. Mutuality may also facilitate authenticity and goal sharing, two key processes associated with relationship flourishing [11,37].

The Romance subscale captures the behavioral and cognitive processes that facilitate love and passion between partners, namely physical intimacy and savoring. Our findings suggest that physical intimacy may help foster lovebird relationships by increasing emotional intimacy, passion, and feelings of love between partners. Indeed, both sexual intimacy and physical touch have been found to play a vital role in relationship functioning and maintenance [38,39,40,41]. The Romance subscale also measures instances of relational savoring within lovebird relationships, which has been shown to buffer the negative effects of relationship stressors on relationship satisfaction, especially for those in highly satisfied couples [42,43,44,45].

The Disconnect subscale is comprised of items that reflect ambivalence and indifference within the relationship, all of which are characteristic of numbed relationships [7]. The Disconnect subscale also illustrates how the absence of positive relationship processes may lead to disaffection, a leading cause of relationship distress and dissolution characterized by a gradual decline in love and increase in feelings of indifference towards one’s partner [46].

Mutuality, Romance, and composite lovebird scores were also associated with less insecure attachment, demonstrating satisfactory discriminant validity [47]. The Disconnect subscale was positively associated with both insecure attachment dimensions. This is not surprising, as we would expect individuals in lovebird couples to exhibit characteristics of secure attachment, such as engaging in constructive interactions (e.g., ability to discuss problems or insecurities), reporting greater feelings of connectedness, and experiencing less general conflict [47]. However, since we did not explicitly measure secure attachment in our study, more research is needed to confirm this.

### 5.2. Affective Experiences in Lovebird Relationships

We also examined the association of the Lovebird Scale with affective state, which is a central tenet of individual flourishing [17,18]. Both lovebird subscales were positively associated with PA. Romance was more strongly associated with PA than Mutuality, which suggests that taking time to think about and enjoy one’s partner appears to be an important mechanism for increasing PA. This makes sense, given that savoring is a self-regulatory process used to generate positive emotions [43,44,45]. Physical intimacy has also been linked to more PA and less NA [48]. Thus, physical intimacy may enhance feelings of closeness between partners, which in turn promotes greater PA within lovebird relationships. Furthermore, composite lovebird scores emerged as significant and unique predictors of PA, above and beyond existing measures of relationship quality (RAS and DAS-32), thereby providing preliminary evidence for the incremental validity of the Lovebird Scale. This finding suggests that the Lovebird Scale may offer additional insight into positive affectivity within romantic relationships over existing measures.

The Disconnect subscale was associated with lower PA and greater NA. This is unsurprising, given that the aspects of numbed relationships, such as ambivalence and relational distancing, have been associated with greater NA [49,50,51]. Further, Gottman’s research also revealed that some couples do not engage in negative, hostile behavior during conflict; rather, they behave in a more detached way, experiencing very little PA during conflict interactions. Though these relationships may last longer than the “disastrous” relationships, the lack of PA within them is more likely to result in relationship dissolution over time [52].

Interestingly, composite lovebird scores were not a unique predictor of NA when accounting for RAS and DAS-32 scores. These results suggest that existing relationship quality scales may be better at tapping into the negative affectivity within relationships than the Lovebird Scale. While surprising, there are several possible explanations for this finding. First, it is possible that people in lovebird relationships may value and validate whatever the partner is feeling, whether it is positive or negative, and there is no particular emphasis on making sure the other person does not experience NA. Second, even though they may experience NA, the higher levels of PA reported in lovebird relationships may “cancel out” the negative emotions to some degree [8]. Couples will inevitably experience NA during conflict; however, highly satisfied couples do not enter these states as often, and when they do, they are able to escape more easily [52]. Third, it is possible that the time to recovery for a given level of NA may be shorter in lovebird relationships. Although there is evidence to suggest that social sharing can lead to momentary increases in NA [53], one study found that sharing daily hassles predicted short- and long-term increases in closeness in romantic partners [54]. However, since there was no significant association in the current sample, we cannot rule out the possibility it was missed with this sample size. Similarly, Fowers, Laurenceau, Penfield, Cohen, Lang, Owenz and Pasipanodya [15] also found that the RFS was a better predictor of positive relationship processes but was less sensitive to measures of relationship distress than existing measures of relationship quality since these previous scales were primarily focused on identifying relationship dysfunction rather than relationship flourishing [7,15,16]. Thus, it should not be surprising that the Lovebird Scale was not a unique predictor of NA in the present study. Future research should examine the types of loving and other positive feelings that characterize lovebird versus numbed relationships, such as being in love, as well as being happy, satisfied, and warm. Longitudinal or daily experience studies can also track this ratio over time to determine how it relates to temporal changes in relationship flourishing.

### 5.3. Theoretical and Conceptual Implications

The Lovebird Scale adds to the growing field of positive relationship science, which emphasizes the role of positive relationship processes that contribute to relationship health and flourishing rather than the presence or absence of negative relationship processes [7]. Second, the Lovebird Scale advances the science of love by operationalizing the construct of “optimal” romantic relationships, going beyond broad ratings of relationship satisfaction to distinguish between lovebirds and other types of committed but numbed relationships. As recent research has noted, there is more to relationship quality than varying degrees of satisfaction, and this is reflected through the development of other measures of relationship flourishing, such as the Relationship Flourishing Scale and the Relational-Connectivity Scale. However, what sets the Lovebird Scale apart from these other measures is that it assesses constructs that are measured by each of those scales as well as includes other relevant domains of relationship flourishing, such as physical intimacy, relational savoring, and responsiveness. Additionally, while any couple may take the Lovebird Scale, the initial scale creation and validation were based on the notion of an extraordinary love for each other. By restricting the range of likely responses by screening for high satisfaction, we were able to more finely distinguish nuances in the items.

Finally, we examined associations between the Lovebird Scale and affective state, which, to our knowledge, has not been addressed by existing relationship flourishing measures. In their paper, Fincham and Beach [7] argue that understanding relationship flourishing requires consideration of positive and negative affectivity and, more specifically, what ratio is needed to create a positivity offset. Continuing to measure affective state within lovebird relationships will provide valuable insight into the everyday affective experiences of flourishing relationships. Further, we argue that the higher levels of PA observed in lovebird relationships occurs because there is a positive feedback loop of love within the couple that, in turn, enables each partner to thrive as both individuals and a relationship partner. In line with theories such as the find-remind-and-bind theory of gratitude [55] and the broaden-and-build theory of positive emotions [56], this positive feedback loop within the couple contributes to both higher levels of relationship quality on the dyad level as well as greater PA on the individual level. More research is needed to demonstrate this positive feedback loop as well as the hypothesized individual thriving of individuals in a lovebird relationship. Longitudinal studies may also help elucidate how the maintenance of lovebird relationships promotes sustained PA over time.

### 5.4. Clinical Implications

In addition to these conceptual implications, the present research has implications for practitioners as well. To date, many relationship interventions have targeted negative processes (e.g., maladaptive communication patterns, conflict resolution) that lead to poor relationship outcomes [57]. The study of lovebird relationships can improve existing couples’ interventions and inspire novel interventions, particularly as it relates to everyday interactions within relationships. Bradbury and Bodenmann [57] argue that there is a need to systematically assess couples’ ability to create, share, and capitalize on positive experiences to determine the best treatment plan for a given couple. The Lovebird Scale would be particularly useful for this, as it aims to capture the behaviors, thoughts, and feelings that contribute to positive relationship experiences in everyday life. For example, if relationship partners report low scores on the Romance subscale, practitioners may recommend interventions that focus on increasing relational savoring or intimacy [43], whereas low scores on the Mutuality subscale may indicate a need to focus on improving communication and increasing trust between partners through behavioral couple therapy approaches [57]. Research on the affective experiences of lovebird relationships would also be useful for developing emotion-focused couple therapy interventions that address both the way lovebirds manage their own emotions as well as the way they manage each other’s emotions. In sum, incorporating the Lovebird Scale into clinical and therapeutic settings has the potential to improve practitioners’ ability help couples overcome acute conflict or reinvigorate relationships that are trending towards committed but numbed relationships.

### 5.5. Limitations and Future Directions

There are several limitations of the present study that should be addressed. First, self-report questionnaires are subject to various biases, such as response bias, social desirability, and positive sentiment override, which refers to when an individual’s perception of specific aspects of the relationship are influenced by their general feelings about the relationship [58]. Second, the cross-sectional nature of this study limits our ability to draw conclusions regarding causality. For example, since data were only collected at one time point, the findings may not reflect relationship quality over time and may be influenced by temporal fluctuations in relationship quality for a given level of PA or NA. Experience sampling or daily diary studies may help illustrate temporal changes in ratings of lovebirdness or numbness, while longitudinal studies would be beneficial for understanding how lovebird relationships are developed and maintained over time. In-laboratory behavioral observations may also help illustrate how lovebird couples interact with each other and how the factors identified in the present research manifest themselves in these interactions. Third, this study only captures the experiences of one relationship partner. Future research should have both partners complete the scale, as this could highlight potential relationship issues if one partner believes they are lovebirds but the other does not.

Another limitation to consider is the study sample. The use of an online recruitment platform such as CloudResearch allowed for us to recruit individuals from across the U.S., but despite this, our sample was still primarily white, female, educated (i.e., bachelor’s degree or higher), and heterosexual, which ultimately limits the generalizability of our findings to more diverse populations. In addition, given the use of online recruitment methods, there is always the possibility that “bots” completed the survey, which can impact the quality of the data [19]. While multiple steps were taken to address this limitation in the current research (i.e., use of data security measures), future research should aim to replicate these findings in different populations and use other forms of data collection to minimize the possibility of automated bot responses.

There are also limitations regarding the scale development process that should be addressed. First, it is possible that the results from the CFAs may have been underpowered due to the small sample size. While the sample sizes in this study met previous recommendations for factor analysis (i.e., greater than 200), other recommendations suggest using a ratio of 10+ per item [22,24]. The small sample sizes may also inflate the risk of Type 1 error and multicollinearity, further underscoring the need for larger sample sizes in future research. Second, because we excluded individuals with low relationship quality from the analyses, the non-normal distribution of the data could have affected the analyses. Third, several double-barreled items were included in the final iteration of the Lovebird Scale, which may have also contributed to the less-than-ideal model fit indices found in Studies 2 and 3. Future research should routinely examine the model fit of the Lovebird Scale in larger samples and propose modifications, as needed, to improve the reliability and validity of these scales in addition to using item response theory (IRT) methods instead of traditional factory analysis methods like those used in the present study.

A fourth methodological limitation to consider is the limited number of measures used to establish the construct validity of the Lovebird Scale. Future research should incorporate more measures of love and other measures relevant to the specific facets of lovebird relationships (e.g., Perceived Responsiveness and Insensitivity Scale, Passionate Love Scale, Eros Love Style Scale, Friendship-Based love Scale, etc.) to further examine the construct validity of the scale.

Another issue to consider is the discriminant and incremental validity of the scale, given that the Lovebird Scale subscales displayed similar correlations with existing measures of relationship quality and affective state. One area for future investigation could be the construct of shared reality, which has been linked to various positive relationship processes such as feeling known and understanding others [59]. We would expect global lovebird scores to correlate more strongly with shared reality compared to existing measures of relationship satisfaction given the Lovebird Scale’s emphasis on thoughts and behaviors that facilitate loving feelings and other positive affective states between partners. We were also not able to fully demonstrate the incremental validity of the Lovebird Scale. Future studies should include existing measures of relationship flourishing, such as the RFS and RCS, to establish the incremental validity of the Lovebird Scale, particularly when it comes to assessing both overall relationship quality as well as affective state.

Future research should also aim to determine if certain behaviors (as measured by self-report or through behavioral coding) are more strongly associated with one subscale over the others. For example, we might expect that individuals who score higher on the Romance subscale display a higher frequency of affectionate touch behaviors or report a greater degree of closeness with their partner, while those who score higher on the Mutuality subscale may report more responsiveness and use more constructive communication styles when discussing a marital conflict. Those who score high on the Disconnect subscale, thus being in more of a numbed relationship, might report feeling less authentic and use less constructive communication styles when discussing a conflict (e.g., stonewalling). Understanding these subscale-specific nuances could be helpful for answering future research questions, such as how specific relationship dynamics, as captured by the Lovebird Scale, predict mental and physical health outcomes in couples. It would also be interesting to examine how the subscales of the Lovebird Scale differ as a function of a relationship length, as this may help differentiate the various subscales from each other given the moderate-to-high correlations between them. Indeed, previous studies have found that the association between various aspects of love (companionate, passionate, romantic) differ among short- and long-term couples [60].

## 6. Conclusions

Despite the methodological limitations mentioned above, the present study provides preliminary evidence for the reliability and construct validity of the Lovebird Scale, a novel assessment of relationship quality that aims to bridge the gap between lay and academic understandings of lovebirds and relationship flourishing by developing a multifaceted scale that captures the specific behaviors, cognitions, and feelings that contribute to flourishing relationships, which differ from other long-term committed but numbed relationships. The Lovebird Scale offers a holistic assessment of romantic relationships by incorporating dimensions that are not fully captured by existing measures and integrating multiple facets, such as romance, mutuality, and disconnection, into a single scale, which allows for a more nuanced exploration of relationship well-being. Furthermore, the Lovebird Scale may also be a useful tool for delineating between those who are moderately satisfied but numbed and those who are highly satisfied with their relationships. By focusing on the factors that contribute to relationship flourishing, researchers and practitioners may be better able to design and implement interventions to help couples not only address issues with relationship functioning but also promote more meaningful, flourishing relationships.

## Figures and Tables

**Table 2:** Results from the exploratory factor analysis in Study 1 ^1^.

Item	Factor Loadings		
	LB-M	LB-R	LB-D
6. I trust my partner completely and I can tell my partner anything.	0.70		
10. My partner never intentionally insults me, puts me down, or makes me feel bad.	0.82		
12. My partner and I have recurring problems that we can’t get past.	−0.55		0.37
13. We are each other’s best friend.	0.57	0.32	
33. We are very kind to each other.	0.78		
38. My partner accepts every part of me, even the things I dislike about myself.	0.69		
41. My partner and I respect each other’s opinions, even when we don’t agree with each other.	0.73		
44. My partner and I fit well together.	0.61	0.25	
45. I support my partner in their goals and aspirations, and they do the same for me.	0.58		
47. I can talk to my partner about anything, even if it is a difficult conversation.	0.86		
48. I don’t have to sacrifice aspects of myself to keep my partner happy.	0.76		
1. When I hear certain songs, I think of how much I love my partner.		0.64	
4. All of life’s ups and downs seem pretty insignificant compared to the love that we share.		0.54	
5. Our sex life is deeply satisfying.		0.69	
17. Sometimes when I’m alone I find myself thinking about how much I love my partner.		0.70	
18. The more time we spend together the more I enjoy my partner’s company.	0.27	0.55	
19. We share a seamless continuum of compassionate and erotic love.		0.76	
22. I find my partner extremely physically attractive.	−0.21	0.70	
24. I often find myself thinking about special things I can do to make my partner happy.		0.68	
30. Touching is natural and fundamental to our relationship.		0.53	
37. My partner and I go through life savoring moments together.	0.31	0.52	
20. I am easily attracted to others when I am away from home.			0.77
27. When I see lovey-dovey couples I think they are unrealistic or out of touch.			0.54
31. We stay together for external reasons such as marriage vows and children, more than because of our enjoyment of being together.	−0.23		0.54
32. I often think about former lovers.			0.87
36. Although I love my partner, I would not say that I am currently “in love”.			0.66
46. I feel like I need space after we spend a lot of time together.			0.60
14. My partner and I know how to make each other laugh, even on our bad days.	0.46	0.29	
26. We don’t have to do anything in particular to thoroughly enjoy being together.	0.44		
34. I am more myself when I am alone than when I am with my partner.	−0.27		0.49
49. There are things about my partner that I wish I could change	−0.25		0.40

^1^ *N* = 540. LB-M = Lovebird Scale—Mutuality subscale; LB-R = Lovebird Scale—Romance subscale; LB-D = Lovebird Scale—Disconnect subscale. Factor loadings were estimated using principal axis factoring with a Promax rotation. Factor loadings less than 0.20 were omitted from the table for clarity purposes.

## Data Availability

All data and analysis code have been made publicly available at the “APA Journal Articles: Data and Related Resources” Open Science Framework repository and can be accessed at https://osf.io/3p69f/. This study’s design and its analysis were not pre-registered. A pre-print of this manuscript can be accessed at https://psyarxiv.com/zavkf.

## Supplementary Materials

The following information can be downloaded at https://www.mdpi.com/article/10.3390/bs14090747/s1, Table S1: Sample characteristics for Studies 1–3.

## Appendix A. The Lovebird Scale

Instructions: You have been asked to complete this questionnaire because you are currently involved in a committed long-term romantic relationship. Please answer the following questions about your relationship using the scale below.

1 = Strongly disagree; 2 = Disagree; 3 = Disagree somewhat; 4 = Neither agree nor disagree; 5 = Agree somewhat; 6 = Agree; 7 = Strongly agree

1. I trust my partner completely and I can tell my partner anything. (m)
2. When I hear certain songs, I think of how much I love my partner. (r)
3. I am easily attracted to others when I am away from home. (d)
4. My partner never intentionally insults me, puts me down, or makes me feel bad. (m)
5. Our sex life is deeply satisfying. (r)
6. We stay together for external reasons such as marriage vows and children, more than because of our enjoyment of being together. (d)
7. We are very kind to each other. (m)
8. Sometimes when I’m alone I find myself thinking about how much I love my partner. (r)
9. I often think about former lovers. (d)
10. My partner accepts every part of me, even the things I dislike about myself. (m)
11. The more time we spend together the more I enjoy my partner’s company. (r)
12. Although I love my partner, I would not say that I am currently “in love”. (d)
13. My partner and I respect each other’s opinions, even when we don’t agree with each other. (m)
14. I find my partner extremely physically attractive. (r)
15. I feel like I need space after we spend a lot of time together. (d)
16. My partner and I fit well together. (m)
17. I often find myself thinking about special things I can do to make my partner happy. (r)
18. I am more myself when I am alone than when I am with my partner. (d)
19. I support my partner in their goals and aspirations, and they do the same for me. (m)
20. Touching is natural and fundamental to our relationship. (r)
21. There are things about my partner that I wish I could change. (d)
22. I can talk to my partner about anything, even if it is a difficult conversation. (m)
23. We are each other’s best friend. (r)
24. My partner and I have recurring problems that we can’t get past. (d)
25. I don’t have to sacrifice aspects of myself to keep my partner happy. (m)
26. My partner and I go through life savoring moments together. (m)

Mutuality = items 1, 4, 7, 10, 13, 16, 19, 22, 25, 26
Romance = items 2, 5, 8, 11, 14, 17, 20, 23
Disconnect = items 3, 6, 9, 12, 15, 18, 21, 24

Scoring: Subscale scores are calculated by taking the average of each set of items. Composite lovebird scores are calculated by first reverse-scoring items on the Disconnect subscale and then taking the average of all items in the scale.

## Appendix B. Relationship Prototypes

Instructions: Please read the statement below and use the sliding scale to indicate how accurately it describes your relationship.

Extremely inaccurate (0) -------- Extremely accurate (100)

### Appendix B.1. Relationship Prototype 1 (Lovebird Prototype; 164 Words)

You are in love with your partner and feel that you have found “the love of your life”, a real soul mate. You thoroughly enjoy being together, and you connect on multiple levels—intellectually, emotionally, and sexually. You frequently tell one another that you love each other. You do everything you can to make each other happy. No relationship is perfect, but you are able to work things out when things go off track because you are able to communicate clearly and respectfully. Keeping this relationship going in a very positive manner does not require a lot of work. You feel that you are experiencing the best that life has to offer and feel very fortunate to be in this relationship. You think the world would be a better place if other couples got along as well as the two of you do.

### Appendix B.2. Relationship Prototype 1 (Numbed Prototype; 167 Words)

You chose to be with your partner for very good reasons, and the two of you are committed to each other for the long term. You respect one another and have worked out a way to have a life together that on the whole is successful. There are many things that you like and even admire about your partner, but there are also some things that bother you a lot, and they are not likely to change. You find yourself attracted to other people and even think about them occasionally but do not take any actions because you are committed to the relationship and don’t want to destabilize it. Relationships can be a lot of work, but all things considered, they are worth it. Two people may initially have a lot of passion for one another, but eventually, that goes away and is replaced by other ways of staying connected. Overall, people shouldn’t rely exclusively on one person for their happiness, so a well-rounded life is essential.
