# Supplementary material for: Development and Preliminary Validation of the Lovebird Scale
Source: Behav Sci (Basel). 2024 Aug 26;14(9):747. doi: 10.3390/bs14090747 (PMC11429147; doi:10.3390/bs14090747)
Supplement: Supplementary file 1 [file behavsci-14-00747-s001.zip › behavsci-3052736-supplementary.pdf]

Supplemental Materials: Development and Preliminary Validation of the Lovebird Scale

Table S1. Sample characteristics for Studies 1-3.[1]

Variable	Study 1 (N = 540)		Study 2 (N = 215)		Study 3 (N = 241)	
	n	%	*n*	%	*n*	%
Sex						
Female	327	60.6	144	67.0	168	69.7
Male	213	39.4	69	32.1	72	29.9
Gender						
Female	322	59.6	143	66.5	166	68.9
Male	210	38.9	68	31.6	71	29.5
Non-binary	5	0.9	0	0	3	1.2
Transgender	1	0.2	3	1.4	1	0.4
Other	2	0.4	1	0.5	0	0
Ethnicity						
White	432	80.0	182	84.7	200	83.0
Black	44	8.1	9	4.2	16	6.6
Asian	22	4.1	8	3.7	12	5.0
Hispanic	19	3.5	8	3.7	10	4.1
Other	23	4.3	8	3.7	3	1.2
Education						
High school diploma or less	63	11.7	21	10.2	30	12.4
Some college	115	21.3	34	15.8	49	20.3
Associates degree	65	12.0	30	14.0	21	8.7
Bachelor's degree	180	33.3	84	39.1	91	37.8
Graduate degree	117	21.6	45	20.9	50	20.7
Sexual Orientation						
Heterosexual	459	85.0	185	86.0	209	86.7
Gay or Lesbian	15	2.8	3	1.4	7	2.9
Bisexual	52	9.6	22	10.2	16	6.6
Other	14	2.6	5	2.4	9	3.7
Relationship Status						
Dating	99	18.3	42	19.5	35	14.5
Engaged	58	10.7	28	13.0	32	13.3
Married	365	67.6	132	61.4	168	69.7
Other	18	3.3	13	6.0	6	2.5
Cohabitating						
Yes	493	91.3	197	91.6	224	92.9
No	47	8.7	18	8.4	17	7.1

[1] Total N = 996. Average age for Studies 1-3 were 39.9(SD = 13.2), 38.7(SD = 11.6), and 39.1(SD = 10.2) years old, respectively. Average relationship length in Studies 1-3 was 11.4(SD = 10.8), 10.5(SD = 12.7), and 11.7(SD = 11.2) years, respectively.
